# Supplementary material for: Integrin activation is an essential component of SARS-CoV-2 infection
Source: Sci Rep. 2021 Oct 14;11:20398. doi: 10.1038/s41598-021-99893-7 (PMC8516859; doi:10.1038/s41598-021-99893-7)
Supplement: Supplementary file 1 — Supplementary Information. [file 41598_2021_99893_MOESM1_ESM.docx]

**Supplemental Material**

**
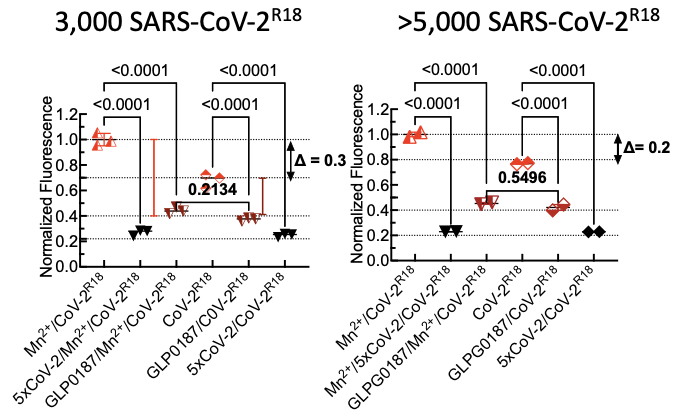
**

**Supplemental Figure 1. Flow cytometry assays of binding inhibition of relatively low and high SARS-CoV-2^R18^ concentration to cells, treated with broad-spectrum RGD antagonist, GLPG0187.** Vero E6 suspension cells in 40 µl volumes (1,000 cells/µl) were first incubated with 10 µM integrin antagonists or 5x unlabeled Sars-CoV-2 (CoV-2) in ±Mn^2+^ media for 20 min at 37°C. Sars-CoV-2^R18^ was then added to the tubes and incubated for another 20 min. The samples were centrifuged and resuspended in 95 µl HHB buffer and analyzed on a flow cytometer ∆ = 0.3 and ∆ = 0.2 refers to the relative differences in site occupancy of Mn^2+^-treated and resting cells in samples with a relatively low stoichiometric ratio of virus versus cells, compared to samples with a higher ratio. All other parameters are conserved.
